# A rationally designed small molecule for identifying an *in vivo* link between metal–amyloid-β complexes and the pathogenesis of Alzheimer's disease†
†Electronic supplementary information (ESI) available: Experimental section, Tables S1–S3, and Fig. S1–S6. See DOI: 10.1039/c4sc03239j



**DOI:** 10.1039/c4sc03239j

**Published:** 2015-01-27

**Authors:** Michael W. Beck, Shin Bi Oh, Richard A. Kerr, Hyuck Jin Lee, So Hee Kim, Sujeong Kim, Milim Jang, Brandon T. Ruotolo, Joo-Yong Lee, Mi Hee Lim

**Affiliations:** a Department of Chemistry , Ulsan National Institute of Science and Technology (UNIST) , Ulsan 689-798 , Republic of Korea . Email: mhlim@unist.ac.kr; b Department of Chemistry , University of Michigan , Ann Arbor , MI 48109-1055 , USA . Email: bruotolo@umich.edu; c Asan Institute for Life Sciences , Asan Medical Center , Seoul 138-736 , Republic of Korea . Email: jlee@amc.seoul.kr; d Department of Neurology , University of Ulsan College of Medicine , Seoul 138-736 , Republic of Korea; e Life Sciences Institute , University of Michigan , Ann Arbor , Michigan 48109-2216 , USA

## Abstract

Multiple factors, including amyloid-β (Aβ), metals, and reactive oxygen species (ROS), are involved in the development of Alzheimer's disease (AD). Metal ions can interact with Aβ species generating toxic oligomers and ROS *in vitro*; however, the involvement of metal–Aβ complexes in AD pathology *in vivo* remains unclear. To solve this uncertainty, we have developed a chemical tool (**L2-b**) that specifically targets metal–Aβ complexes and modulates their reactivity (*i.e.*, metal–Aβ aggregation, toxic oligomer formation, and ROS production). Through the studies presented herein, we demonstrate that **L2-b** is able to specifically interact with metal–Aβ complexes over metal-free Aβ analogues, redirect metal–Aβ aggregation into off-pathway, nontoxic less structured Aβ aggregates, and diminish metal–Aβ-induced ROS production, overall mitigating metal–Aβ-triggered toxicity, confirmed by multidisciplinary approaches. **L2-b** is also verified to enter the brain *in vivo* with relative metabolic stability. Most importantly, upon treatment of 5XFAD AD mice with **L2-b**, (i) metal–Aβ complexes are targeted and modulated in the brain; (ii) amyloid pathology is reduced; and (iii) cognition deficits are significantly improved. To the best of our knowledge, by employing an *in vivo* chemical tool specifically prepared for investigating metal–Aβ complexes, we report for the first time experimental evidence that metal–Aβ complexes are related directly to AD pathogenesis.

## Abstract 2

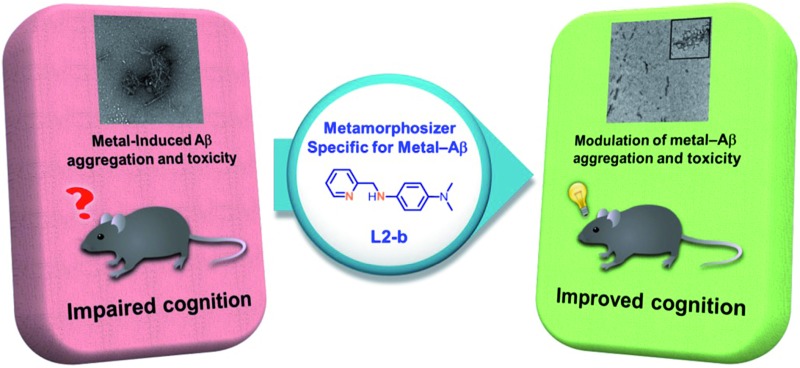
An *in vivo* chemical tool designed to target metal–Aβ complexes and modulate their activity was applied to the 5XFAD mouse model of Alzheimer’s disease (AD) demonstrating the involvement of metal–Aβ in AD pathology.

## Introduction

Alzheimer's disease (AD), a progressive neurodegenerative disease, is the most common form of dementia afflicting 24 million people worldwide.^1^ Despite AD being the sixth leading cause of death in the United States, there are currently no disease modifying treatments; approved therapies only offer symptomatic relief without having an effect on the underlying pathogenesis.^1,2^ Development of effective therapeutics has been hindered by the fact that AD pathogenesis is still poorly understood. Pathologically, AD is characterized by the accumulation of aggregated, misfolded proteins, such as amyloid-β (Aβ) peptides (two major forms exist, Aβ_40_ and Aβ_42_).^3,4^ The amyloid cascade hypothesis suggests that Aβ is the causative agent in AD;^5^ however, the etiology of AD can be multifactorial; of particular interest is the role of Aβ with other factors (*i.e.*, metals) toward AD development.^4,6–11^

High concentrations of Fe, Cu, and Zn (*ca.* low mM) are found within Aβ deposits in *ex vivo* tissues from the AD-afflicted brain.^12,13^ These metal ions are observed to coordinate to Aβ peptides *in vitro* forming metal–Aβ complexes which could direct toxicity *via* two possible pathways:^4,6–11,14–19^ (i) metals could influence the Aβ aggregation pathways leading to the generation and stabilization of toxic Aβ oligomers;^4,7–9,14^ (ii) redox active metal ions (*i.e.*, Cu(i/ii) and Fe(ii/iii)) associated with Aβ are shown to produce reactive oxygen species (ROS) under physiological conditions through Fenton-like reactions.^4,6–11,16–19^ Overproduction of ROS by metal–Aβ can result in oxidative stress and eventually neuronal death in the AD-affected brain. Although the reactivity of metal–Aβ (*i.e.*, (i) metal–Aβ aggregation (toxic Aβ oligomer formation) and (ii) redox active metal–Aβ-triggered ROS generation, *vide supra*) has been indicated *in vitro*,^4,6–11,14–19^ the direct involvement of metal–Aβ complexes in AD pathogenesis *in vivo* is uncertain.

Metal chelating agents have shown that the interference of metal–Aβ interactions as well as the modulation of metal distribution in the brain could lead to an improvement in AD pathology.^4,19–24^ 8-Hydroxyquinoline derivatives have been employed to regulate metal-related neurotoxicity in AD; some small molecules, including clioquinol (**CQ**) and **PBT2**, have indicated promising results for possible AD treatment in clinical trials.^4,22,23^ The effects of **CQ** and **PBT2** are mainly from their ability to act as an ionophore to redistribute metal ions in the brain instead of directly disrupting metal–Aβ complexes;^4,19,23–25^ thus, these compounds would not be able to directly probe the relation between metal–Aβ complexes and AD pathogenesis. Therefore, chemical tools, termed as metamorphosizers, have been recently developed in order to (i) specifically target metal–Aβ complexes and (ii) alter the interaction between the metal and Aβ, consequently (iii) redirecting the toxic aggregation pathway of metal–Aβ into off-pathway, less toxic unstructured Aβ forms and (iv) reducing metal–Aβ-induced ROS production, which eventually alleviates metal–Aβ-linked toxicity.^4,24^

**Fig. 1:**
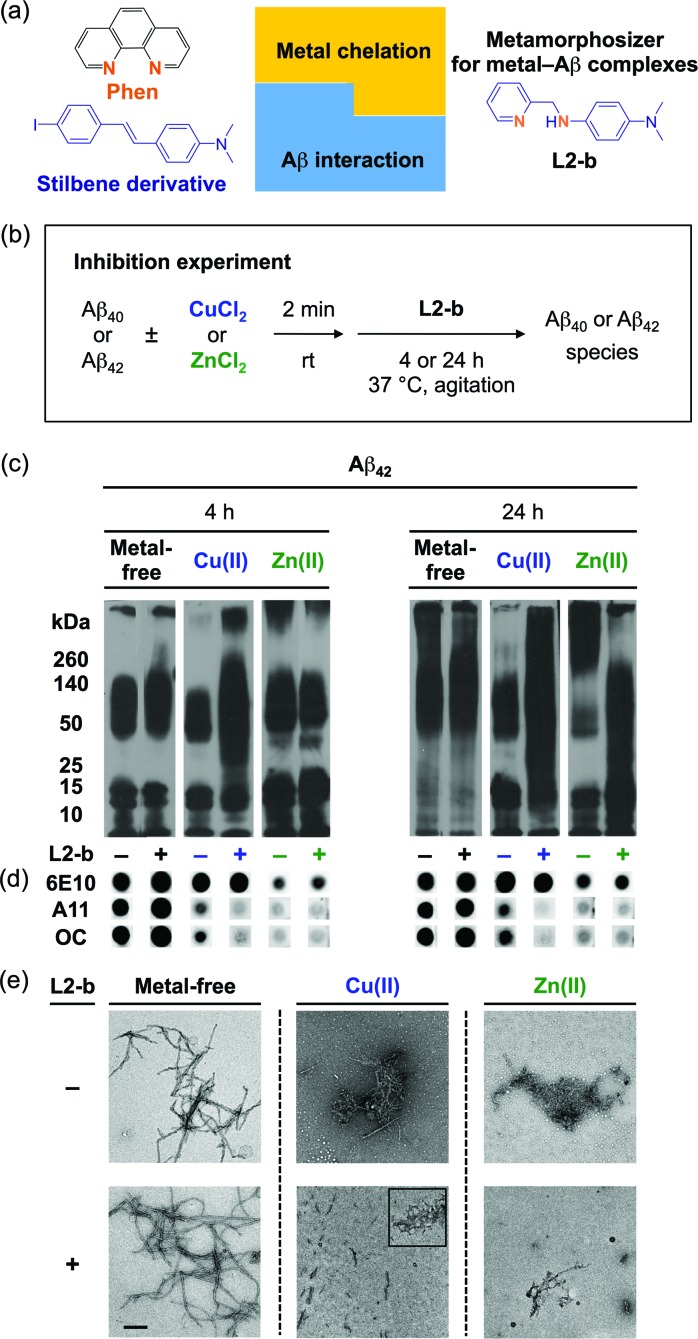
Design principle of **L2-b** and its effect on metal-free and metal-induced Aβ aggregation. (a) Design principle of **L2-b**, a metamorphosizer for metal–Aβ complexes: a metal binding site (orange) is incorporated into an Aβ interacting framework (blue). (b) Scheme showing the inhibition experiment: metal-free or metal-treated [CuCl_2_ (blue) or ZnCl_2_ (green)] Aβ_40_/Aβ_42_ was incubated with (+) or without (–) **L2-b** for 4 h (left) and 24 h (right). Conditions: [Aβ] = 25 μM; [Cu(ii) or Zn(ii)] = 25 μM; [**L2-b**] = 50 μM; pH 6.6 (for Cu(ii) samples) or pH 7.4 (for metal-free and Zn(ii) samples); 37 °C; constant agitation. (c) Analysis of the size distribution of the resultant Aβ_42_ species by gel electrophoresis and Western blotting with an anti-Aβ antibody (6E10). (d) Dot blot analysis of the resulting Aβ_42_ species employing 6E10, an anti-Aβ oligomer antibody (A11), and an anti-Aβ fibril antibody (OC). (e) TEM images of the 24 h incubated samples (scale bar = 200 nm).

Herein, we demonstrate that a chemical tool (**L2-b**, Fig. 1a) stands out as being well suited *in vivo* for identifying the association of metal–Aβ_40_/Aβ_42_ with AD pathogenesis, through *in vitro* biochemical/biophysical/cytotoxicity/metabolism investigations, as well as *in vivo* brain uptake studies. Our *in vivo* tool specifically interacts with metal–Aβ over metal-free Aβ and generates a ternary **L2-b**–metal–Aβ complex causing structural compaction, as validated by mass spectrometry (MS) and ion mobility-mass spectrometry (IM-MS). Most significantly, we present the first report that the control of metal–Aβ interaction and reactivity by an *in vivo* chemical tool mitigates amyloid pathology and improves cognitive deficits in the 5XFAD AD mouse model. This robust AD mouse model develops severe amyloid pathology and cognitive decline at an early age through high expression of three familial mutant types of human amyloid precursor protein (hAPP; Swedish, Florida, and London) and two mutant forms of presenilin (PSEN1; M146L and L286V).^26^ Overall, our studies establish strong experimental evidence for an *in vivo* link between metal–Aβ and AD development, implying that targeting metal–Aβ complexes could be an effective strategy for the future development of new therapeutics.

## Results and discussion

### Design principle and characterization of a chemical tool for investigating metal–Aβ complexes *in vivo*

**L2-b** (Fig. 1a) was designed to target metal–Aβ complexes and modulate their interaction/reactivity with subsequent reduction of toxicity,^27^ in order to determine whether they are connected with AD pathology. For *in vivo* applications, first, chemical tools for this purpose must have specificity toward metal–Aβ complexes in order to limit the disruption of other metalloproteins.^4,24^ This property can be imparted into small molecules by using inorganic chemistry concepts to allow specificity for disease-relevant metal ions (Fe(ii/iii), Cu(i/ii), and Zn(ii)), along with limiting the metal binding affinity (*K*_d_) to ≥10^–10^ M, and by including structural components for Aβ interaction.^4,24^ To satisfy this aspect, **L2-b** (a bidentate ligand; Fig. 1a) was constructed upon incorporation of two nitrogen donor atoms (for metal chelation) into the structure of an Aβ aggregate imaging agent (stilbene derivative; for Aβ interaction),^28^ which could interact with metal–Aβ complexes (Fig. 1a).^27^ **L2-b** is shown to have apparent *K*_d_ values of *ca.* 10^–10^ and 10^–6^ M for Cu(ii) and Zn(ii), respectively, and is relatively selective for Cu(ii) over other biologically relevant bivalent ions.^27^ Secondly, the blood–brain barrier (BBB) permeability of **L2-b** is critical for applications in the brain, which was first predicted by considering Lipinski's rules of drug-likeness and observing calculated log BB values.^27^ Employing CD1 mice, *in vivo* brain uptake studies of **L2-b** newly confirmed its BBB penetration. **L2-b** (*ca.* 250 ng g^–1^) is observed to be available in the brain when administered by oral gavage (10 mg kg^–1^) to the mice (Table S1†). Thirdly, the metabolic stability of **L2-b** for *in vivo* applications was also verified utilizing human liver microsomes. Susceptibility of **L2-b** to metabolism is between 30 min and 120 min indicating that this compound has moderate metabolic stability, suggesting its suitability for use *in vivo*. Lastly, **L2-b** acts as an antioxidant as well as an inhibitor of Cu(i/ii)– or Cu(i/ii)–Aβ-induced ROS production as presented in previous studies.^27,29^ From our newly performed study using the Trolox equivalent antioxidant capacity in a cellular environment (*i.e.*, murine neuroblastoma Neuro-2a (N2a) cell lysates),^30^ **L2-b** exhibits a greater free radical scavenging capacity (2.3 ± 0.2) than Trolox (1.0 ± 0.1), a known antioxidant vitamin E analogue. Therefore, **L2-b** is clearly demonstrated to be viable for *in vivo* use as a chemical tool for exploring the association of metal–Aβ complexes with AD pathogenesis.

### Specific modulation of metal-induced over metal-free Aβ aggregation pathways *in vitro*

To elucidate whether **L2-b** could redirect metal–Aβ aggregation into off-pathway amorphous Aβ aggregates, suggested to be less toxic or nontoxic,^31^ while leaving metal-free Aβ cases unaffected, inhibition (Fig. 1b) and disaggregation (Fig. S1a†) experiments^30^ were performed employing Aβ_40_ and Aβ_42_, the two main Aβ forms found in the AD-affected brain. The influence of **L2-b** on both metal-free and metal-mediated Aβ aggregation was monitored at short and long incubation time points.^32^ Gel electrophoresis and Western blotting (gel/Western blot, utilizing an anti-Aβ antibody, 6E10)^30^ were conducted to determine the molecular weight (MW) distribution of the resulting Aβ aggregates. Dot blot analysis with an anti-Aβ oligomer antibody A11 ^33^ and an anti-Aβ fibril antibody OC,^34^ along with 6E10, was carried out to identify the type of Aβ species produced. Moreover, transmission electron microscopy (TEM) images were taken to visualize the morphologies of the resultant Aβ aggregates.^30^

Both the inhibition and disaggregation experiments indicate that **L2-b** does not modulate the aggregation pathways of both Aβ_40_ and Aβ_42_ under metal-free conditions after either short or long incubation periods. Nearly identical MW distributions of the Aβ species in the absence and presence of **L2-b** were observed in the gel/Western blots (Fig. 1c, S1b, and S2a†). The dot blots of the inhibition samples indicated A11 (oligomer)- and OC (fibril)-positive aggregates for metal-free Aβ_40_/Aβ_42_ even when treated with **L2-b** (Fig. 1d and S2b†). TEM images revealed that Aβ fibrils were mainly present in both the inhibition and disaggregation experiments of metal-free Aβ_40_/Aβ_42_ with and without **L2-b** after 24 h of incubation (Fig. 1e, S1c, and S2c†). Thus, metal-free Aβ aggregation is not noticeably influenced upon treatment with **L2-b**.

In contrast to the metal-free conditions, significantly noticeable changes in the metal [Cu(ii) or Zn(ii)]-induced Aβ_40_ and Aβ_42_ aggregation pathways by **L2-b** were observed compared to **L2-b**-untreated analogues. In both the inhibition and disaggregation experiments, after 24 h of incubation of the Cu(ii)–Aβ species with **L2-b**, the resulting peptide species with a wide range of MWs were visualized by gel/Western blot (Fig. 1c, S1b, and S2a†). In the inhibition studies of both Aβ_40_ and Aβ_42_, as well as in the disaggregation experiment of Aβ_42_, Cu(ii)–Aβ samples treated with **L2-b** even for 4 h also exhibited the distinct MW distribution of Aβ (Fig. 1c, S1b and S2a†). Distinguishably, **L2-b** was capable of limiting the formation of A11- and OC-positive Cu(ii)-induced Aβ_40_/Aβ_42_ aggregates at both short and longer incubation times (Fig. 1d and S2b†). Morphologies of **L2-b**-incubated Cu(ii)–Aβ, analyzed by TEM, displayed both narrower and shorter fibrils, as well as unstructured Aβ aggregates in the inhibition experiments (Fig. 1e and S2c†); while less dense, thinner fibrils were mainly observed in the disaggregation experiments (Fig. S1c†). In the case of Zn(ii)–Aβ, **L2-b** could also transform the aggregation pathways (Fig. 1c, S1b, and S2a†). The TEM studies revealed **L2-b**-triggered, smaller amorphous Zn(ii)–Aβ aggregates in both the inhibition and disaggregation experiments (Fig. 1e, S1c, and S2c†). Overall, **L2-b** is observed to redirect metal–Aβ aggregation mainly into unstructured Aβ aggregates that are generated *via* the off-pathway aggregation and are known to be less toxic or nontoxic.^31^ Thus, **L2-b** could be used as a chemical tool specific for such anti-amyloidogenic activity toward metal–Aβ complexes over metal-free Aβ in this manner.

### Formation of structurally-compact complexes with metal–Aβ not metal-free Aβ *in vitro*

**Fig. 2:**
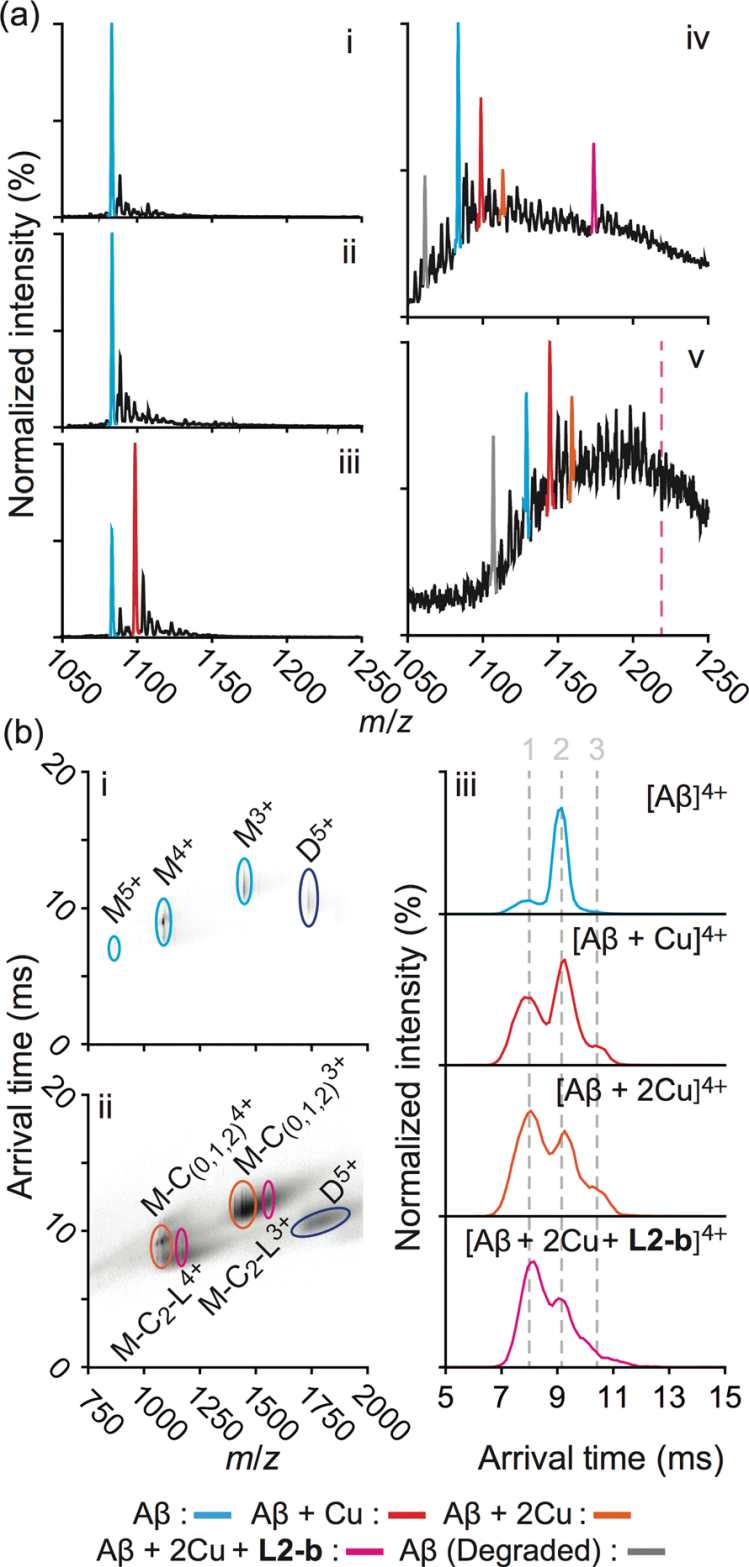
Mass spectrometric (MS) and ion mobility-mass spectrometric (IM-MS) analyses of Aβ in the presence of **L2-b** and/or Cu(ii). (a) Comparison of incubated Aβ 4+ charge states in the samples containing (i) Aβ_40_ (18 μM) alone and Aβ_40_ co-incubated with (ii) excess **L2-b** (120 μM), (iii) Cu(ii) (40 μM), and (iv) both **L2-b** (40 μM) and Cu(ii) (40 μM) [(v) Aβ_42_ (pH 9, 18 μM) with **L2-b** (40 μM) and Cu(ii) (40 μM) is also presented]. Consistent with data shown here, the gray signal represents a currently unidentified chemical modification of the N-terminus up to, and including, residue 5 (Fig. S3†) but not F4 (Fig. S4†). The projected location of the complex consisting of Aβ_42_, Cu(ii), and **L2-b** in a ratio of 1 : 2 : 1 is indicated in pink. (b) IM-MS analysis of Aβ_40_ (18 μM) incubated in the (i) absence and (ii) presence of **L2-b** (40 μM) and Cu(ii) (40 μM). Extracted arrival time distributions support the existence of three resolvable structural populations (Collision Cross Section (CCS) data, Table S2†).

In order to explore the specific interaction of **L2-b** with metal–Aβ over metal-free Aβ, nanoelectrospray ionization-MS (nESI-MS) studies were employed (Fig. 2a). When metal-free Aβ_40_ was allowed to react with **L2-b**, no binding events were observed, even with a six fold excess of the ligand (Fig. 2a(ii)). In comparison, incubating a comparatively smaller concentration of **L2-b** with Aβ_40_ and Cu(ii) promoted readily observed levels of complexes containing Aβ_40_, Cu(ii), and **L2-b** approximately in the ratio 1 : 2 : 1, supporting the metal specific nature of the interaction (Fig. 2a(iv)). The formation of a ternary complex between **L2-b** and Cu(ii)–Aβ_40_ is supported by the previously reported NMR studies of **L2-b** with Zn(ii)–Aβ_40_ in solution.^27^

Additionally, another MS signal was observed. This signal corresponds to an intact molecular mass of 89.24 Da less than the full-length Aβ_40_ peptide in good agreement with ternary Aβ_40_–Cu(ii)–**L2-b** complex formation (gray, Fig. 2a(iv)). Tandem MS data (Fig. S3†) and subsequent analysis of the fragment ions indicate that this new signal corresponds to a chemical modification within the first five residues of Aβ_40_ (D_1_A_2_E_3_F_4_R_5_). Given a mass measurement error of ±1 Da, and supporting **L2-b** binding experiments performed using an Aβ_40_ F4A sequence variant, as well as acetylated analogs (Fig. S4†), we can eliminate alterations to F4 as a source of the product observed and show that free primary amines are critical for binding and subsequent Aβ degradation. While no direct observations of the Cu(ii)–**L2-b**-bound Aβ_42_ form were indicated by MS, the 89.24 Da mass loss product was detected (gray, Fig. 2a(v)) upon addition of both **L2-b** and Cu(ii) to the samples, implying the generation of a transient ternary Aβ_42_–Cu(ii)–**L2-b** complex of unknown stoichiometry. These Aβ_40_/Aβ_42_ fragmentation results also suggest that, as expected, Cu(ii) likely binds to Aβ proximal to the site of **L2-b** attachment.^4,7^ In all cases, neither **L2-b** nor Cu(ii) was detected in complex with the identified Aβ degradation product. Detailed structures of these ternary complexes will be the subject of future studies.

To study the molecular level structural dynamics by which **L2-b** redirects metal–Aβ aggregation pathways, IM-MS experiments of the complexes produced were performed. A comparison of the arrival time distributions of the metal-free Aβ_40_ form with the different ligated states supports an increasing level of structural compaction as additional components (*i.e.*, Cu(ii) and **L2-b**) associate with Aβ_40_ (Fig. 2b and Table S2†). Analyzing the arrival time distributions for all complex states presented, along with the nESI-MS data, our IM-MS investigations demonstrate that **L2-b** is capable of specifically interacting with Cu(ii)-bound Aβ over metal-free Aβ, subsequently promoting a high level of structural compaction of the complex. This binding of **L2-b** to metal–Aβ with increased structural compactness could be a key property for the distinguishable reorganization of metal–Aβ aggregation pathways, similar to the previously suggested molecular level mode of action of EGCG toward metal–Aβ complexes which promotes the generation of nontoxic unstructured aggregates *via* off-pathway aggregation.^24,31^

### Targeting and reacting with metal–Aβ complexes in living cells and in the brain of 5XFAD AD mice

**Fig. 3:**
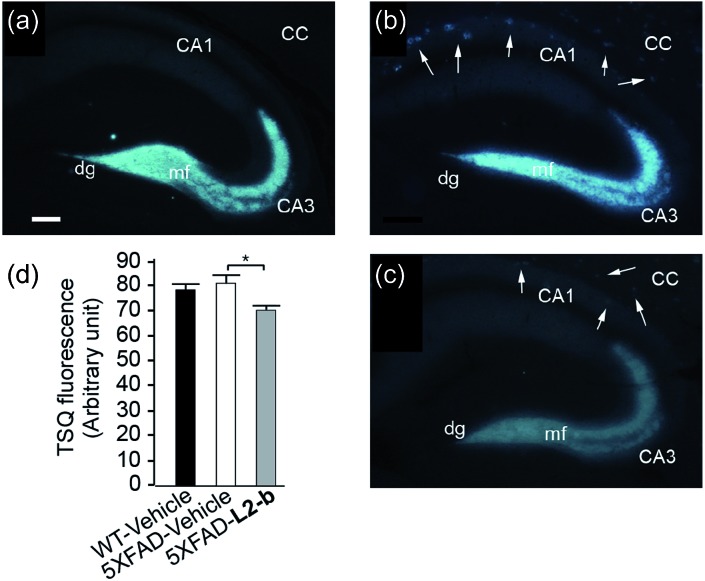
Levels of Zn(ii) in the brain tissues of nontransgenic wild type (WT) and 5XFAD transgenic mice. Amounts of Zn(ii) were determined using a fluorescent dye, 6-methoxy-(8-*p*-toluenesulfonamido)quinoline (TSQ), in the mossy fiber region (mf) of brains from (a) WT and (b and c) 5XFAD transgenic mice after intraperitoneal administration of (a and b) the vehicle or (c) **L2-b** (1 mg per kg per day) for three weeks beginning at three months of age (scale bar = 100 μm). The fluorescence response of TSQ was also shown in the zone of amyloid plaques in 5XFAD mice (shown by arrows; b and c). CC, corpus callosum; CA, cornu amonis; dg, dentate gyrus. (d) The fluorescence intensity of TSQ was quantified in the mossy fiber region (mf in a–c) of vehicle-treated WT (black bar; *n* = 6) and 5XFAD male mice (white bar; *n* = 10), or **L2-b**-treated 5XFAD male mice (gray bar; *n* = 9), where the measurement was performed using five sagittal sections selected randomly from each animal and denoted as an arbitrary unit of the TSQ fluorescence [mean ± standard error of the mean (S.E.M)]. *P < 0.05 by one-way analysis of variance (ANOVA).

The effect of **L2-b** on metal–Aβ_40_/Aβ_42_-induced toxicity was first examined using N2a cells, as an indication of its interaction with metal–Aβ complexes. An increase (*ca.* 10–20%) in cell viability for both Aβ_40_ and Aβ_42_ was displayed upon treatment of cells incubated with Cu(ii) or Zn(ii), Aβ, and **L2-b** (10 μM each; Fig. S5†). Moving forward, the ability of **L2-b** to penetrate the BBB and interact with metal–Aβ species in the brain was verified in the 5XFAD AD mouse model. Zn(ii) found in Aβ plaques was visualized in the brain tissue slices by a fluorophore specific for Zn(ii), 6-methoxy-(8-*p*-toluenesulfonamido)quinolone (TSQ; Fig. 3).^35^ Administration of **L2-b** to 5XFAD AD mice intraperitoneally for three weeks on a daily basis starting at the age of three months resulted in drastically diminished fluorescence of TSQ in the plaques (arrows shown in Fig. 3c). Additionally, in the hippocampal mossy fiber terminals, a Zn(ii)-rich region in the brain,^36^ there was no difference in the fluorescence of TSQ between wild type and 5XFAD AD mice treated daily with the vehicle, whereas **L2-b** reduced fluorescence by *ca.* 13% (*P* < 0.05) in 5XFAD AD mice over the same time span (Fig. 3d). Thus, these *in vivo* studies suggest that **L2-b** is BBB permeable and can enter the brain to interact with intracerebral metals, including those found in Aβ plaques.

### Reduction of amyloid pathology in 5XFAD AD mice

To identify the direct involvement of metal–Aβ complexes in amyloid pathology leading to improved cognition, the 5XFAD mouse model of AD^26^ was chosen. **L2-b** (1 mg kg^–1^) was injected into nontransgenic littermates (wild type) and 5XFAD AD mice *via* the intraperitoneal route for three weeks on a daily basis starting at the age of three months. All mice survived the consecutive treatments, which rarely caused changes in body weight (Table S3†). Necropsy of all major organs in **L2-b**-treated mice revealed no gross changes.

**Fig. 4:**
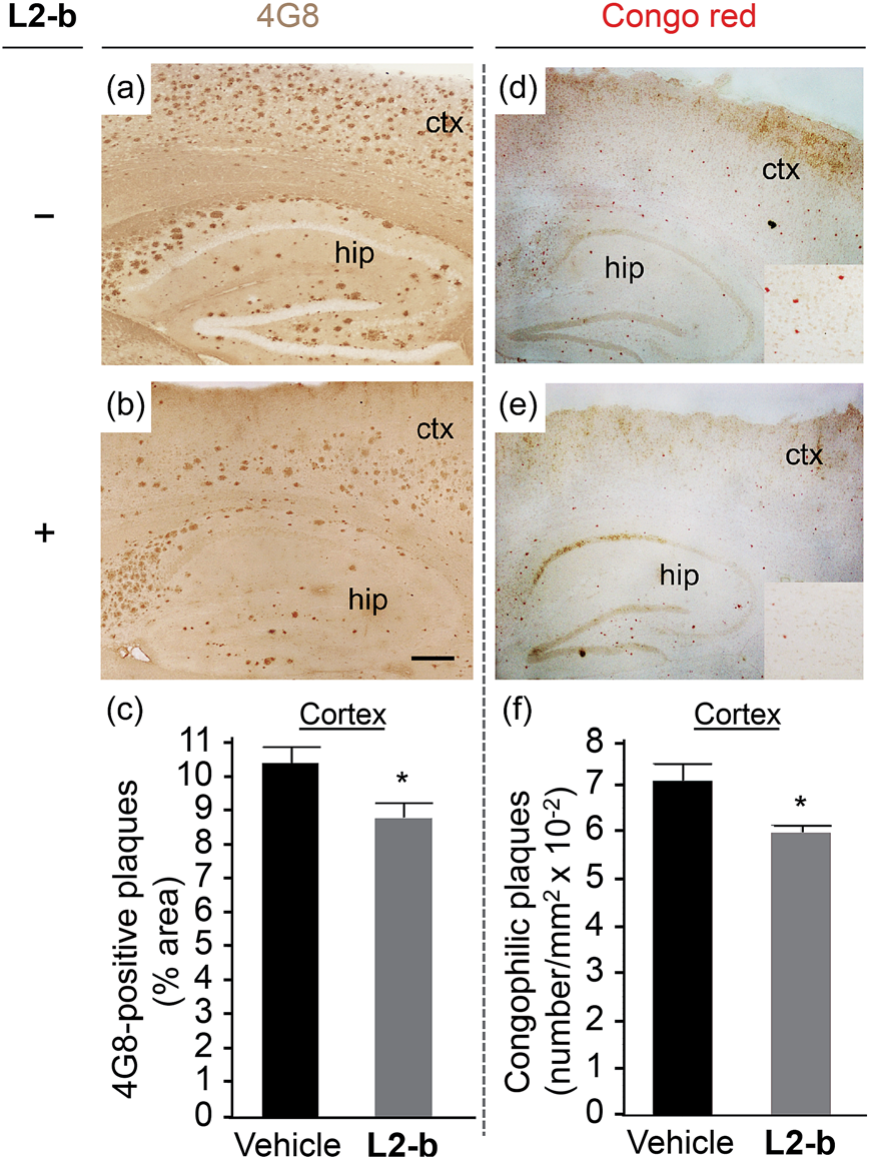
Effect of daily treatments with **L2-b** for three weeks on the amyloid deposits in the brains of 5XFAD male mice. Representative microscopic images of (a and b) 4G8-immunostained or (d and e) Congo red stained brain sections of 5XFAD mice, which were given daily (a and d) the vehicle or (b and e) **L2-b** (1 mg per kg per day) *via* intraperitoneal injection for three weeks starting at three months of age (magnification = 40×; scale bar = 100 μm). Inset in (d) and (e): enlarged micrographs of congophilic amyloid plaques in the cortical area (magnification, 400×; hip, hippocampus; ctx, cortex). To evaluate the amyloid pathology of the vehicle (black bars; *n* = 5)- or **L2-b** (gray bars; *n* = 7)-treated male 5XFAD mice, (c) the load of 4G8-immunoreactive amyloid deposits and (f) the number of congophilic amyloid plaques in the cortex were measured in five brain sections taken from each animal. *P < 0.05 by one-way ANOVA.

**Fig. 5:**
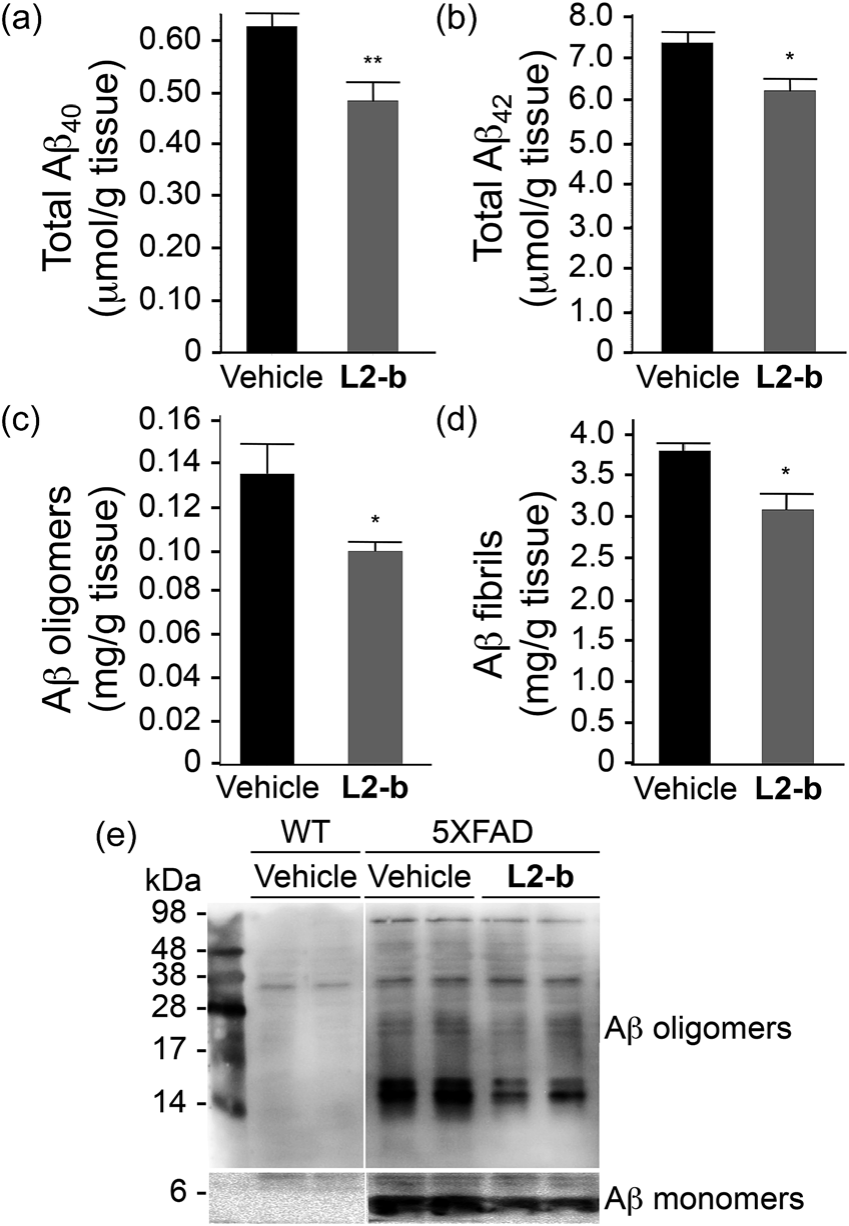
Levels of Aβ in whole brain tissues of three-month-old male 5XFAD mice. The amounts of (a) total Aβ_40_, (b) total Aβ_42_, (c) PBS-soluble Aβ oligomers, and (d) Aβ fibrils were assessed using ELISA after three weeks of treatment with vehicle (black bars; *n* = 5) or **L2-b** (1 mg per kg per day; gray bars; *n* = 7). Bars denote the levels of Aβ, which were calculated from three independent experiments and expressed as values per gram of tissue. *P < 0.05 or **P < 0.01 by one-way ANOVA. (e) 4–20% (lower panels) and 16.5% (upper panels) tris–glycine gel/Western blot analyses were performed to visualize the Aβ monomers and aggregates, respectively, in the brain tissue lysates of wild type (WT; left panels) and 5XFAD male mice (right panels).

The potential association of **L2-b** with amyloid pathology was investigated by first observing the amyloid plaque load in the brain tissue of 5XFAD AD mice. When the brain tissue slices of **L2-b**-administered 5XFAD AD mice were stained with an APP/Aβ-specific antibody (4G8) or a compact core amyloid plaque indicator (Congo red), it was found that the amyloid plaque burden was ameliorated (Fig. 4). Reduction (*ca.* 15%) of both the area of 4G8-immunoreactive deposits and the number of congophilic amyloid plaques was revealed in the cortex of **L2-b**-treated 5XFAD AD mice when compared to vehicle-treated 5XFAD AD mice (Fig. 4c and f). The changes in the amount of both Aβ_40_ and Aβ_42_ in the brain tissues of 5XFAD AD mice following **L2-b** administration were also assessed. Total amounts of Aβ peptides were analyzed by an enzyme-linked immunosorbent assay (ELISA) in sodium dodecyl sulfate (SDS)- and formic acid (FA)-soluble brain tissue lysates (Fig. 5a and b and S6†), as well as oligomeric and fibrillar Aβ aggregates in the phosphate buffered saline (PBS)-soluble fraction (Fig. 5c and d).^37^ Relative to vehicle-treated 5XFAD mice, the **L2-b**-treated 5XFAD mice showed diminished cerebral levels of both Aβ_40_ and Aβ_42_ in all fractions (*ca.* 15–20%, *P* < 0.05, Fig. 5a and b). Oligomeric and fibrillar Aβ species in the PBS fraction were additionally abated by 27% and 15%, respectively (*P* < 0.05, Fig. 5c and d). Similarly, the overall reduction of Aβ species was also indicated by gel/Western blot, where Aβ monomers and oligomers were noticeably decreased in brain tissue lysates from **L2-b**-treated 5XFAD AD mice (Fig. 5e). Together, these studies demonstrate that daily administration of **L2-b** to the AD model mitigates amyloid pathology in AD, including the load of amyloid plaque deposits and the levels of a wide range of conformations from monomers to fibrils.

### Cognitive improvement in 5XFAD AD mice

**Fig. 6:**
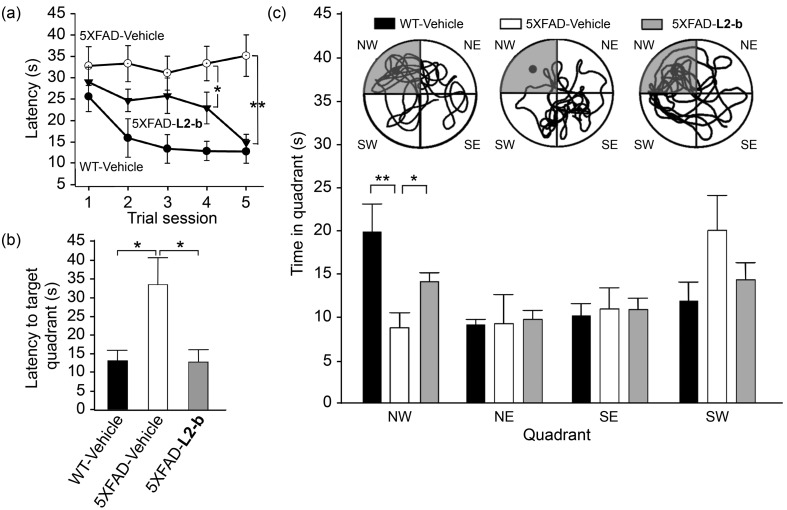
Learning and memory abilities of three-month-old male wild type (WT) and 5XFAD male mice treated with vehicle (black and white bars) and **L2-b** (gray), measured using the Morris water maze task. (a) The escape latency time was counted every day during the period of the 21^st^–25^th^ daily treatments of either vehicle or **L2-b** and the probe trials were performed on the day of the final 25^th^ treatment to measure (b) how quickly the mice reach and (c) how long they spend in the target quadrant (NW, highlighted in gray; circles show images of the representative tracks of the mice in the water maze). *P < 0.05 or **P < 0.01 by one-way ANOVA (*n* = 6, 13, and 14 for vehicle-treated WT and vehicle-/**L2-b**-treated 5XFAD mice, respectively).

Investigation of behavioral performance was carried out by administering **L2-b** to 5XFAD AD mice which suffer from deficits in learning and memory capabilities as amyloid pathology progresses.^26^ The Morris water maze was used to evaluate different aspects of spatial learning and memory in three-month-old 5XFAD AD mice.^26^ The wild type mice, which were consecutively injected with vehicle during the experimental period, normally took shorter times upon repetition of the training trial to find the escape platform, located in the northwest (NW) quadrant (Fig. 6a and b). In contrast, vehicle-treated 5XFAD AD mice spent longer times searching for and reaching the platform indicating they had difficulties with learning and memory (Fig. 6). Administration of **L2-b** to 5XFAD AD mice led to a remarkable improvement in the performance of the task. **L2-b**-treated 5XFAD AD mice were capable of finding the target in a comparable time to the wild type mice displaying significantly better memory and learning abilities than their untreated 5XFAD AD littermates (*P* < 0.05, Fig. 6a and b). Additionally, **L2-b**-treated 5XFAD AD mice took a more direct and easier path than the vehicle-treated 5XFAD AD mice to search for the platform (*P* < 0.05, Fig. 6c). Therefore, **L2-b**, a chemical reagent specific for metal–Aβ, ameliorates cognitive defects in the AD mouse model, along with the attenuation of amyloid pathology. These overall *in vivo* observations and results indicate that metal–Aβ complexes could be directly linked to AD pathogenesis.

## Conclusions

In summary, for the first time, experimental evidence affirms that metal–Aβ complexes can be directly associated with AD pathogenesis, by applying the first *in vivo* chemical tool which specifically targets metal–Aβ complexes and ameliorates metal–Aβ reactivity (*i.e.*, metal–Aβ aggregation, formation of toxic oligomers, and ROS production) in 5XFAD AD mice. Our findings presented herein demonstrate the feasibility of developing small molecules as *in vivo* chemical tools for studying metal–Aβ. In addition, our studies indicate that research efforts toward understanding metal–Aβ-induced pathological pathways and identifying interrelated partners with metal–Aβ in AD onset and progression at the molecular level should continue to be made. The current and future outcomes, obtained from metal–Aβ-involved AD research, can open new directions for our long-term goal, the discovery of effective drugs for this fatal neurological disorder.
