# Supplementary material for: A rationally designed small molecule for identifying an in vivo link between metal–amyloid-β complexes and the pathogenesis of Alzheimer's disease
Source: Chem Sci. 2015 Jan 27;6(3):1879–86. doi: 10.1039/c4sc03239j (PMC5494539; doi:10.1039/c4sc03239j)
Supplement: Supplementary file 1 [file SC-006-C4SC03239J-s001.pdf]

Electronic Supplementary Material (ESI) for Chemical Science.
This journal is © The Royal Society of Chemistry 2015

Supporting Information

for

A Rationally Designed Small Molecule for Identifying an *In Vivo* Link of Metal–Amyloid-β Complexes to the Pathogenesis of Alzheimer's Disease

Michael W. Beck,[§ab] Shin Bi Oh,[§c] Richard A. Kerr,[b] Hyuck Jin Lee,[ab] So Hee Kim,[c] Sujeong Kim,[c] Milim Jang,[a] Brandon T. Ruotolo,*[b] Joo-Yong Lee*[cd] and Mi Hee Lim*[ae]

[a] Department of Chemistry, Ulsan National Institute of Science and Technology (UNIST) 689-798, Ulsan, Republic of Korea

[b] Department of Chemistry, University of Michigan, Ann Arbor, Michigan 48109-1055, USA

[c] Asan Institute for Life Sciences, Asan Medical Center, Seoul 138-736, Republic of Korea

[d] Department of Neurology, University of Ulsan College of Medicine, Seoul 138-736, Republic of Korea

[e] Life Sciences Institute, University of Michigan, Ann Arbor, Michigan 48109-2216, USA

[§] These authors contributed equally.

* To whom correspondence should be addressed: bruotolo@umich.edu, jlee@amc.seoul.kr, and mhlim@unist.ac.kr

Experimental Section

Materials and Methods. All reagents were purchased from commercial suppliers and used as received unless otherwise noted. Aβ_{40} and Aβ_{42} (the sequence of Aβ_{42}: DAEFRHDSGYEVHHQKLVFFAEDVGSNKGAIIGLMVGGVVIA) were purchased from Anaspec Inc. (Fremont, CA, USA). Compound **L2-b** was prepared using the previously reported procedures.[1] Trace metals were removed from buffers and solutions used in Aβ experiments (*vide infra*) by treating with Chelex overnight (Sigma-Aldrich, St. Louis, MO, USA). Optical spectra for the measurement of Aβ concentrations were recorded on an Agilent 8453 UV-visible (UV-vis) spectrophotometer. Absorbance values for biological assays, including cell viability and antioxidant assays, were measured on a Molecular Devices SpectraMax 190 microplate reader (Sunnyvale, CA, USA).

Aβ Aggregation Experiments. Aβ experiments were performed according to previously published methods.[1-5] Prior to experiments, Aβ_{40} or Aβ_{42} was dissolved in ammonium hydroxide (NH_4OH; 1% v/v, aq). The resulting solution was aliquoted, lyophilized overnight, and stored at −80 °C. A stock solution of Aβ was then prepared by dissolving lyophilized peptide in 1% NH_4OH (10 μL) and diluting with ddH_2O. The concentration of the solution was determined by measuring the absorbance of the solution at 280 nm (ε = 1450 $M^{-1}cm^{-1}$ for Aβ_{40}; ε = 1490 $M^{-1}cm^{-1}$ for Aβ_{42}). The peptide stock solution was diluted to a final concentration of 25 μM in Chelex-treated buffered solution containing HEPES (20 μM, pH 6.6 for Cu(II) samples; pH 7.4 for metal-free and Zn(II) samples) and NaCl (150 μM). For the inhibition studies, **L2-b** (final concentration 50 μM, 1% v/v DMSO) was added to the sample of Aβ (25 μM) in the absence and

presence of a metal chloride salt ($CuCl_2$ or $ZnCl_2$; 25 μM) followed by incubation at 37 °C with constant agitation for 4 or 24 h. For the disaggregation studies, Aβ with and without a metal chloride salt was incubated for 24 h at 37 °C with constant agitation to generate preformed Aβ aggregates. The resulting samples were then treated with **L2-b** (50 μM) and incubated with constant agitation for additional 4 or 24 h.

Gel Electrophoresis and Western Blotting. The samples from the inhibition and disaggregation experiments were analyzed by gel electrophoresis followed by Western blotting using an anti-Aβ antibody (6E10) following previously established procedures.[1-5] Samples (10 μL) were separated on a 10-20% Tris-tricine gel (Invitrogen, Grand Island, NY, USA). Following separation, the proteins were transferred onto nitrocellulose membranes and blocked with bovine serum albumin (BSA, 3% w/v, Sigma-Aldrich, St. Louis, MO, USA) in Tris-buffered saline (TBS) containing 0.1% Tween-20 (TBS-T) for 2 h at room temperature or overnight at 4 °C. The membranes were incubated with an anti-Aβ antibody (6E10, 1:2000, Covance, Princeton, NJ, USA) in a solution of 2% BSA (w/v in TBS-T) for 4 h at room temperature or overnight at 4 °C. After washing with TBS-T (3x, 10 min), a horseradish peroxidase-conjugated goat antimouse secondary antibody (1:5000 in 2% BSA w/v in TBS-T; Cayman Chemical Company, Ann Arbor, MI, USA) was added for 1 h at room temperature. The ThermoScientific SuperSignal West Pico Chemiluminescent Substrate (Thermo Scientific, Rockford, IL, USA) was used to visualize protein bands.

Dot Blot Analysis. Dot blots were performed following previously established

procedures with slight modifications.[6] These experiments were prepared by placing 2.5 μL of the samples from inhibition and disaggregation experiments on nitrocellulose membranes. The samples were allowed to dry and then blocked overnight at 4 °C in 3% BSA w/v in TBS containing 0.01% Tween-20 (dilute TBS-T). The membranes were then incubated with either an anti-Aβ antibody (6E10, 1:2000), an anti-Aβ oligomer antibody (A11, 1:5000 in 2% BSA w/v in dilute TBS-T; Invitrogen), or an anti-Aβ fibril antibody (OC, 1:5000 in 2% BSA w/v in dilute TBS-T; Millipore, Temecula, CA, USA) for 2 h at 4 °C followed by washing with dilute TBS-T (3x, 7 min). The addition of a horseradish peroxidase-conjugated goat antimouse secondary antibody for 6E10-treated membranes or a horseradish peroxidase-conjugated antirabbit secondary antibody (1:10000 in 2% BSA w/v in dilute TBS-T; Promega, Madison, WI, USA) for A11- and OC-treated membranes was subsequently followed. After incubating with the secondary antibody for 1 h at 4 °C, the membranes were washed with dilute TBS-T (3x, 7 min). The Biosesang ECL Plus kit (Biosesang, Gyeonggi-do, Korea) was used to visualize the results on a ChemiDoc MP Imaging System (Bio-Rad, Hercules, CA, USA).

Transmission Electron Microscopy (TEM). Samples for TEM were prepared according to a previously reported method using glow-discharged grids (Formar/Carbon 300-mesh, Electron Microscopy Sciences, Hatfield, PA, USA).[1-5] Images from each sample were taken on a JEM-2100 Transmission Electron Microscope (JEOL Ltd., Tokyo, Japan).

Ion Mobility–Mass Spectrometry (IM–MS). All IM-MS experiments were carried out on

a Synapt G2 (Waters, Milford, MA).[7,8] Samples were ionized using a nano-electrospray source operated in positive ion mode. MS instrumentation was operated at a backing pressure of 2.7 mbar and sample cone voltage of 40 V. Aliquots of Aβ peptides (final concentration 18 μM) were sonicated for 10 sec prior to preincubation with, or without, a source of Cu(II) (copper(II) acetate) at 37 °C for 10 min. After preincubation, samples were treated with or without **L2-b** at 37 °C for 50 min prior to analysis. Solution conditions were 100 mM ammonium acetate (pH 7.5, unless otherwise stated) with 1% v/v DMSO. Acetylation of $A\beta_{40}$ peptides was carried out using methods described previously,[9] prior to overnight dialysis into 100 mM ammonium acetate (pH 7.5). Collision cross-section (CCS) measurements were externally calibrated using a database of known values in helium, using values for proteins that bracket the likely CCS and ion mobility values of the unknown ions.[10,11] CCS values are the mean average of a minimum of five replicates (maximum of eight), with errors reported as the least square analysis output for all measurements. This least square analysis combines inherent calibrant error from drift tube measurements (3%), the calibration R^2 error and two times the replicate standard deviation error. All other conditions are consistent with previously published methods.[3]

Cell Viability Measurements. Murine neuroblastoma Neuro-2a (N2a) cell line was purchased from the American Type Cell Collection (ATCC, Manassas, VA, USA). The cell line was maintained in media containing 45% Dulbecco's modified Eagle's medium (DMEM; Gibco, Life Technologies, Grand Island, NY, USA) and 45% OPTI-MEM Reduced Serum Media (Gibco), supplemented with 9% fetal bovine serum (FBS;

Atlanta Biologicals, Flowery Branch, GA, USA), 1% non-essential amino acids (NEAA), 2 mM glutamine, 100 U/mL penicillin, and 100 mg/mL streptomycin (Gibco). The cells were grown in a humidified atmosphere with 5% CO_2 at 37 °C. For the MTT assay, N2a cells were seeded in a 96 well plate (15,000 cells per 100 μL). The cells were treated with Aβ alone (10 μM), [Aβ + **L2-b** (10 μM, 1% v/v final DMSO concentration)], [Aβ + $CuCl_2$ or $ZnCl_2$ (10 μM)], or [Aβ + $CuCl_2$ or $ZnCl_2$ + **L2-b**]. The N2a cells were also incubated with a metal chloride salt ($CuCl_2$ or $ZnCl_2$; 10 μM), **L2-b** (10 μM), or metal/**L2-b** (1:1 metal:ligand ratio). After 24 h incubation, 25 μL of MTT (Sigma-Aldrich; 5 mg/mL in PBS, pH 7.4; Gibco) was added to each well, and the plate was incubated for 4 h at 37 °C. Formazan produced by the cells was solubilized by addition of an acidic solution of *N,N*-dimethylformamide (50% v/v) and sodium dodecyl sulfate (SDS; 20% w/v, aq) overnight at room temperature in the dark. The absorbance was measured at 600 nm by a microplate reader. Cell viability was calculated relative to that of cells containing an equivalent amount of DMSO. Error bars were calculated as standard errors from three independent experiments.

Antioxidant Assay. The antioxidant activity of **L2-b** was determined by the Trolox equivalent antioxidant capacity (TEAC) assay employing cell lysates following the protocol of an antioxidant assay kit purchased from Cayman Chemical Company with modifications employing the N2a cell line.[2] Cells were seeded in a 6 well plate and grown to approximately 80-90% confluence. Cell lysates were prepared following the previously reported method with modifications.[12] N2a cells were washed once with cold PBS (pH 7.4; Gibco) and harvested by gently pipetting off adherent cells with cold PBS.

The cell pellet was generated by centrifugation (2,000 x g for 10 min at 4 °C). This cell pellet was sonicated on ice (5x for 5 sec pulses with 20 sec intervals between each pulse) in 2 mL of cold assay buffer (5 mM potassium phosphate, pH 7.4, containing 0.9% NaCl and 0.1% glucose). The cell lysates were centrifuged at 5,000 x g for 10 min at 4 °C. The supernatant was removed and stored on ice until use. 10 μL of the supernatant was delivered followed by addition of compound, metmyoglobin, 2,2'-azino-bis(3-ethylbenzothiazoline-6-sulphonic acid (ABTS), and H_2O_2 in the specified order to sample wells on a 96 well plate. After 5 min incubation at room temperature on a shaker, absorbance values at 750 nm were recorded. The percent inhibition was calculated according using the measured absorbance (% inhibition = $(A_0 - A)/A_0$, where A_0 is the absorbance of the supernatant of cell lysates) and was plotted as a function of compound concentration. The TEAC value of ligands was calculated as a ratio of the slope of the standard curve of the compound to that of Trolox (Sigma-Aldrich; Trolox = 6-hydroxy-2,5,7,8-tetramethylchroman-2-carboxylic acid; dissolved in DMSO). Duplicate measurements were conducted in three different experiments.

Brain Uptake Studies. Brain uptake experiments were carried out using male CD1 mice (purchased from Vital River Laboratory Animal Technology Co. Ltd., Beijing, China) by Contract Research Organization, Shanghai ChemPartner Co., Ltd. (Shanghai, China). The studies reported here adhere to the principles of the Association for Assessment and Accreditation of Laboratory Animal Care (AAALAC) International. **L2-b** (10 mg/kg, single dose in sterile water) was administrated to mice by oral gavage. At 30 min postdose (n = 3 at each time point), 150 μL of blood was withdrawn *via* retro

orbital puncture or cardiac puncture and transferred into tubes with spray-coated K_2EDTA as an anticoagulant. Blood samples were put on ice and centrifuged to obtain plasma samples (2000 g, 5 min, 4 °C). Immediately following blood collection, mice were euthanized by pure CO_2 inhalation. The whole brain was collected, rinsed with cold saline, dried on filter paper, and weighed. The brain samples were immediately homogenized with three volumes (v/w) of homogenizing solution (PBS). Both plasma and brain samples were added with an internal standard (propranolol) in acetonitrile (CH_3CN; protein precipitation). The mixture was vortexed for 2 min and centrifuged at 14,000 rpm for 5 min and an aliquot of the supernatant was analyzed for concentration of **L2-b** by LC-MS/MS (UPLC/MS-MS API-5500, Framingham, MA, USA), with the analytical lower limit of quantitation (LLOQ) values for **L2-b** at 2 ng/mL (plasma), 8 ng/mL (brain), and 30 ng/mL (CSF). The supernatant was stored at −80 °C prior to analysis.

Metabolic Stability. The susceptibility of **L2-b** to metabolism was determined by a Contract Research Organization (Shaghai ChemPartner Co., Ltd) using **L2-b** (1 mM) and ketanserin (1 mM; as a reference) in human liver microsomes (0.75 mg/ml) for 0 min, 5 min, 15 min, 30 min, 45 min, 60 min, 75 min, 90 min, and 120 min. The reaction mixtures also contained potassium phosphate buffer (100 mM, pH 7.4) and reduced nicotinamide adenine dinucleotide phosphate (NADPH; 6 mM). Metabolic reactions were initiated by the addition of NADPH and stopped at designated time points by the addition of CH_3CN (135 μL). Precipitate was removed by centrifugation. Supernatant (50 μL) was transferred to a 96 well plate containing 50 μL of millipore water for LC/MS

analysis.

Animals and Drug Administration. Animal studies using male 5XFAD AD model mice were performed in accordance with the Guidelines for Laboratory Animal Care and Use of the Asan Institute for Life Sciences, Asan Medical Center (Seoul, Korea), where they were given free access to chow and drinking water under a 12 h light/dark cycle. 5XFAD transgenic mice overexpress mutant human APP_{695} [K670N/M671L (Swedish), I716V (Florida), and V717I (London)] and PSEN1 (M146L and L286V); thus, the mice rapidly develop pathological features of AD, such as intraneuronal and extracellular Aβ deposition, neurodegeneration, and behavioral disabilities.[13] In this study, each mouse was given an injection with freshly prepared vehicle (1% v/v DMSO in 20 mM HEPES, pH 7.4, 150 mM NaCl) or **L2-b** (1 mg/kg of body weight) into the lower right or left quadrant of their abdomen every day for three weeks using Ultra-Fine™ II insulin syringes (Becton Dickinson, Franklin Lakes, NJ, USA). The body weight of the animal was measured immediately before the injection. Three hours after the final injection, the mice were sacrificed under deep anesthesia. A necropsy was performed to evaluate if there was drug-induced damage and the brain tissues were quickly collected and frozen with liquid nitrogen and stored at –80°C.

Tissue Preparation. The right brain hemisphere of the mice was quickly frozen with liquid nitrogen for biochemical analyses. The left hemispheres were sagittally dissected at the thickness of 12 μm on a cryostat (HM550; Microm, Walldorf, Germany), mounted onto 1% poly-L-lysine-coated glass slides, and thereafter used for histological

evaluations.

Measurement of Synaptic Zn(II). Freshly prepared brain sections (12 μm thickness) were stained with *N*-(6-methoxy-8-quinolyl)-*p*-toluenesulfonamide (TSQ; 4.5 μM, Invitrogen) in 100 μL of 140 mM sodium barbital/sodium acetate buffer (pH 10.0) for 90 sec.[14] After rinsing briefly in physiological saline (0.9% NaCl, pH 7.2), fluorescence of TSQ on the sections was photographed under a fluorescence microscope (Eclipse 80i, Nikon) with a 100X Plan Fluor lens and a UV-2A filter (dichroic, 400 nm; excitation, 330-380 nm; barrier, 420 nm; Nikon) using a digital camera (DS-Fi1/DS-U2; Nikon) and computer-assisted imaging software (NIS-Elements F; Nikon). The mean fluorescence intensity of TSQ in the mossy fiber area was measured using a computer-assisted image analysis program (Image-Pro Plus; Media Cybernetics, Silver Spring, MD, USA) and the level of synaptic Zn(II) was determined by subtracting background fluorescence as obtained at an area outside of the tissue section.

$A\beta_{40}$/$A\beta_{42}$ Quantification. The amounts of $A\beta_{40}$/$A\beta_{42}$ were measured in the brain according to the methods as described previously.[15] Briefly, the protein homogenate fractions were collected in PBS (pH 7.4) containing Complete™ Protease Inhibitor cocktail (Roche Diagnostics, Mannheim, Germany), in 2% SDS (aq), and then in 70% formic acid (FA) by serial centrifugations. The EC buffer-diluted protein fractions were subjected to ELISA using the human $A\beta_{40}$/$A\beta_{42}$ ELISA kit (Invitrogen), where FA-fractions were neutralized with 1 M Tris (pH 11.0) prior to the dilution. In addition, the amounts of the aggregated or oligomeric Aβ in PBS fractions were also measured

using Aβ Oligomers ELISA kit (82E1-specific; IBL International, Hamburg, Germany). The colorimetric quantifications were determined at 450 nm with the Synergy H1 Hybrid microplate reader (BioTek, Winooski, VT, USA), and the cerebral $A\beta_{40}/A\beta_{42}$ amount was calculated as moles per gram of wet brain tissue.

Quantification of Aβ Deposition. In order to evaluate the development of extracellular Aβ deposits, immunohistochemistry studies were performed on the sagittal brain sections using an anti-human Aβ(17-24) antibody (4G8, 1:1000; Covance, Princeton, NJ, USA). After being immunologically reacted with 4G8 and biotynylated anti-mouse secondary antibody (Vector Laboratories, Burlingame, CA, USA), the tissue sections were developed in PBS containing 0.015% diaminobenzidine and 0.001% H_2O_2 (Vector Laboratories) and then examined or photographed under a light microscope (Eclipse 80i; Nikon, Tokyo, Japan). Next, the congophillic amyloid plaques were detected by staining the tissues with Accustain® Congo Red amyloid staining solution (Sigma-Aldrich, St. Louis, MO, USA). The loads of amyloid deposits in the cortex were given as the percent area of 4G8-immunoreactive deposits or the number of congophilic plaques per mm^2 of cortex area.

Immunoblot Analysis of Aβ. Immediately after the brains were collected from the mice, the tissue lysates were prepared in PBS (pH 7.4) containing Complete™ Protease Inhibitor Cocktail (Roche Diagnostics) and stored in liquid nitrogen. The protein amount was measured using a bicinchoninic acid assay (Bio-Rad). Boiled proteins were separated with the sample buffer (62.5 mM Tris, pH 6.8, 2% SDS, 10% glycerol, 0.01%

bromophenol blue, 5% mercaptoethanol, and 50 mM dithiothreitol) on 4-20% or 16.5% Precise Tris-Glycine Gel (Thermo Fisher Scientific, Rockford, IL, USA) and then transferred onto polyvinylidene difluoride membranes (Merck Millipore, Billerica, MA, USA) using semidry blotters (TE70 PWR; Amersham Biosciences, Uppsala, Sweden). After blocking in TBS-T buffer with 5% skimmed milk and 1% BSA (Bovostar; Bovogen, Melbourne, Australia), the blots were reacted with an anti-Aβ antibody (6E10, 1:1000; Covance) and then with horseradish peroxidase-conjugated secondary antibody (1:5000). Immunoreactive proteins were detected using Immobilon Western Chemiluminescent HRP Substrate (Merck Millipore) on the Davinch-Chemi® Chemiluminescence Imaging System (CAS-400SM; CoreBio, Seoul, Korea).

Behavioral Evaluation. Spatial learning and memory abilities were assessed using the Morris water maze task, which was composed of a cylindrical platform (15 cm in diameter) submerged 0.5 cm below the surface of opaque water at the center of a target quadrant in the circular pool. The mice were subjected to three training trials per day to swim and locate the hidden platform for a maximum of 60 sec. The task was conducted at 3 h after each drug injection over a period of five consecutive days starting on the day of the 21st injection, during which vehicle or **L2-b** was still administered. The time and swimming track taken to reach the escape platform were recorded and analyzed on SMART Video Tracking System (Harvard Apparatus, Holliston, MA, USA). Three hours later, the mice entered water again to swim in the absence of the platform for 60 sec and the time spent in each quadrant area was collected.

Statistics. All values are presented as the means ± standard errors of the mean (SEMs) unless otherwise noted. Statistical analysis was performed using one-way analysis of variance (ANOVA) with Student-Newman-Keuls *post hoc* test, or the unpaired *t*-test. Differences with *P* values < 0.05 were considered significant.

References

1 J.-S. Choi, J. J. Braymer, R. P. R. Nanga, A. Ramamoorthy and M. H. Lim, *Proc. Natl. Acad. Sci. USA* 2010, **107**, 21990-21995.

2 M. G. Savelieff *et al*., *Chem. Commu.* 2014, **50**, 5301-5303.

3 S. Lee *et al*., *J. Am. Chem. Soc.* 2014, **136**, 299-310.

4 S.-J. Hyung *et al*., *Proc. Natl. Acad. Sci. USA* 2013, **110**, 3743-3748.

5 Y. Liu *et al*., *Inorg. Chem.* 2013, **52**, 8121-8130.

6 R. Kayed *et al*., *Science* 2003, **300**, 486-489.

7 K. Giles, J. P. Williams and I. Campuzano, *Rapid Commun. Mass. Spectrom.* 2011, **25**, 1559-1566.

8 Y. Zhong, S.-J. Hyung and B. T. Ruotolo, *Analyst* 2011, **136**, 3534-3541.

9 G. T. Hermanson, *Bioconjugate Techniques Second Ed.*; Academic Press: London, United Kingdom, 2008; p 1323.

10 B. T. Ruotolo, J. L. P. Benesch, A. M. Sandercock, S.-J. Hyung and C. V. Robinson, *Nat. Protoc.* 2008, **3**, 1139-1152.

11 M. F. Bush, Z. Hall, K. Giles, J. Hoyes, C. V. Robinson and B. T. Ruotolo, *Anal. Chem.* 2010, **82**, 9557-9565.

12 V. A. Spencer, J.-M. Sun, L. Li and J. R. Davie *Methods* 2003, **31**, 67-75.

13 H. Oakley *et al*., *J. Neurosci.* 2006, **26**, 10129-10140.

14 J.-Y. Lee, J. S. Kim, H.-R. Byun, R. D. Palmiter and J.-Y. Koh, *Brain Res.* 2011, **1418**, 12-22.

15 S. B. Oh *et al*., *Neurobiol. Aging* 2014, **35**, 511-519.

Table S1 Distribution of **L2-b** in male CD1 mice after its administration by oral gavage.

Brain (ng/g)	CSF (ng/mL)	Plasma (ng/mL)	Brain-to-plasma ratio
253 ± 175	289 ± 126	1347 ± 671	0.19 ± 0.16

Table S2 Calculated collision cross section (CCS) values* of the 4+ species of Aβ_{40}.

Species	Conformation 1 (Å^2)	Conformation 2 (Å^2)	Conformation 3 (Å^2)
Aβ_{40}	656.25 ± 29.01	720.35 ± 24.36	782.02 ± 23.86
Aβ_{40} + Cu(II)	663.53 ± 26.70	728.28 ± 29.17	790.08 ± 26.96
Aβ_{40} + 2Cu(II)	664.00 ± 33.33	729.18 ± 24.11	785.98 ± 33.51
Aβ_{40} + 2Cu(II) + **L2-b**	669.26 ± 25.92	722.04 ± 23.77	794.39 ± 47.19

*CCS values are the mean average of a minimum of five replicates (maximum of eight). Errors are reported as the CCS least square analysis output for all replicates.[11]

Table S3 Changes in body weight in nontransgenic littermates and 5XFAD mice during the period of vehicle or **L2-b** treatment.

	Wild type (WT)	5XFAD	5XFAD
Treatment	Vehicle	Vehicle	**L2-b**
Number of animals	6	15	16
Day	Body weight (g) on day of treatment*		
1	21.3 ± 0.5	20.5 ± 0.6	20.8 ± 0.4
2	21.8 ± 0.5	20.3 ± 0.6	20.9 ± 0.4
3	21.3 ± 0.6	20.3 ± 0.5	20.6 ± 0.4
7	21.2 ± 0.7	20.4 ± 0.5	20.5 ± 0.5
14	21.3 ± 0.8	20.6 ± 0.5	20.6 ± 0.5
21	22.2 ± 0.7	21.1 ± 0.4	21.1 ± 0.5

* mean ± S.E.M.

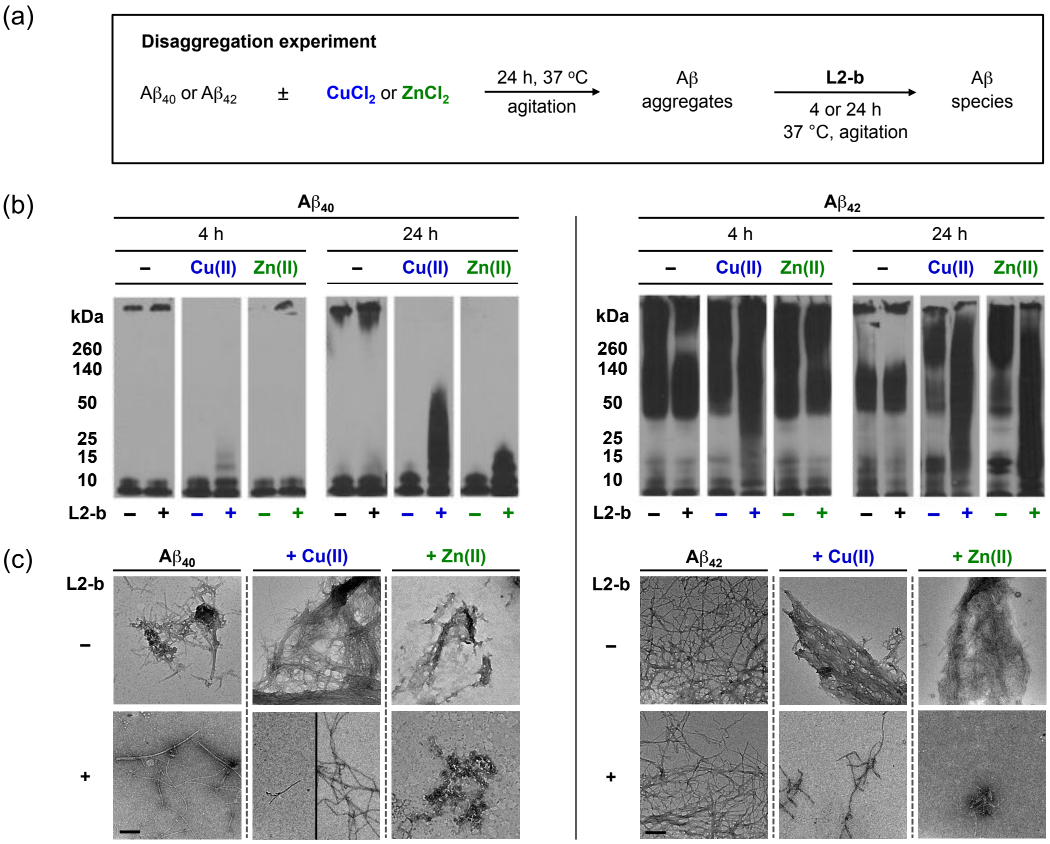

Fig. S1 Effect of **L2-b** on preformed aggregates of $A\beta_{40}$ and $A\beta_{42}$. (a) Scheme of the disaggregation experiment: $A\beta_{40}$ (left) or $A\beta_{42}$ (right) aggregates, generated by 24 h incubation of peptides with and without $CuCl_2$ (blue) or $ZnCl_2$ (green), were treated with **L2-b** (+) followed by an additional incubation of 4 h or 24 h. Conditions: [Aβ] = 25 μM; [Cu(II) or Zn(II)] = 25 μM; [**L2-b**] = 50 μM; pH 6.6 (for $CuCl_2$ samples) or pH 7.4 (for metal-free and Zn(II) samples); 37 °C; constant agitation. (b) Analysis of the size distribution of the resultant Aβ species by gel electrophoresis and Western blotting with an anti-Aβ antibody (6E10). (c) TEM images of the 24 h incubated samples (scale bar = 200 nm).

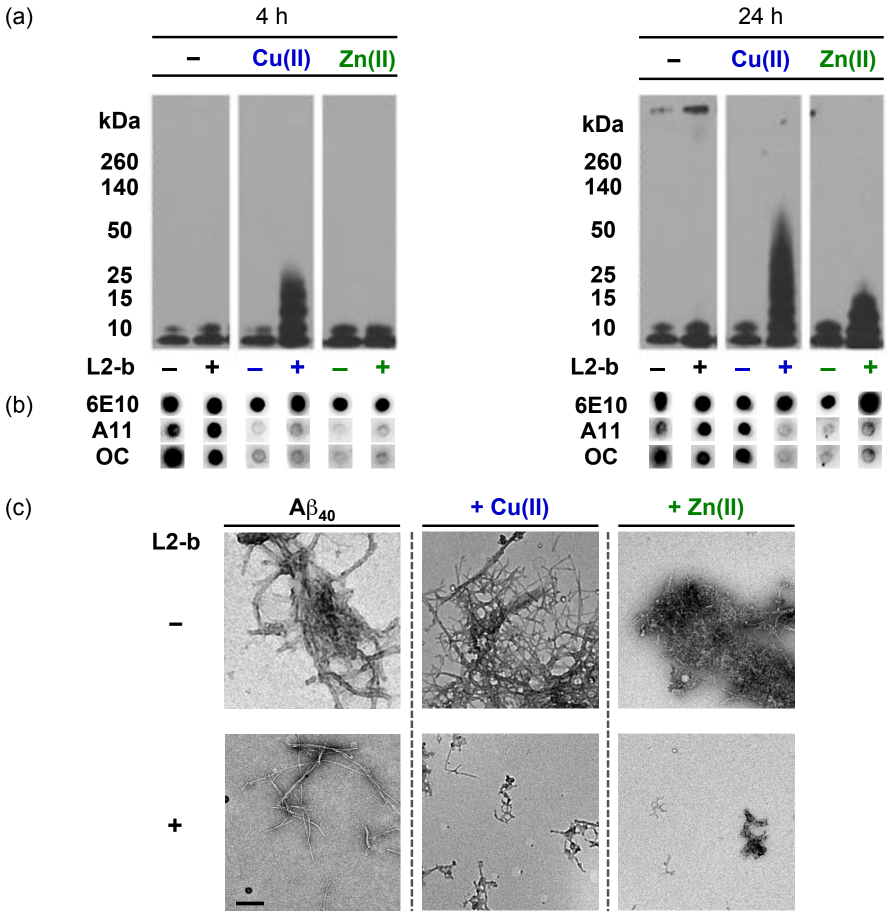

Fig. S2 Effect of **L2-b** on the formation of metal-free and metal-induced Aβ_{40} aggregation (inhibition experiment). The experimental conditions are described in Fig. 1b. (a) Analyses of the size distribution of the resultant Aβ_{40} species by gel electrophoresis and Western blotting with an anti-Aβ antibody (6E10). (b) Dot blot analysis of the resulting Aβ_{40} species employing 6E10, an anti-Aβ oligomer antibody (A11), and an anti-Aβ fibril antibody (OC). (c) TEM images of the 24 h incubated samples (scale bar = 200 nm).

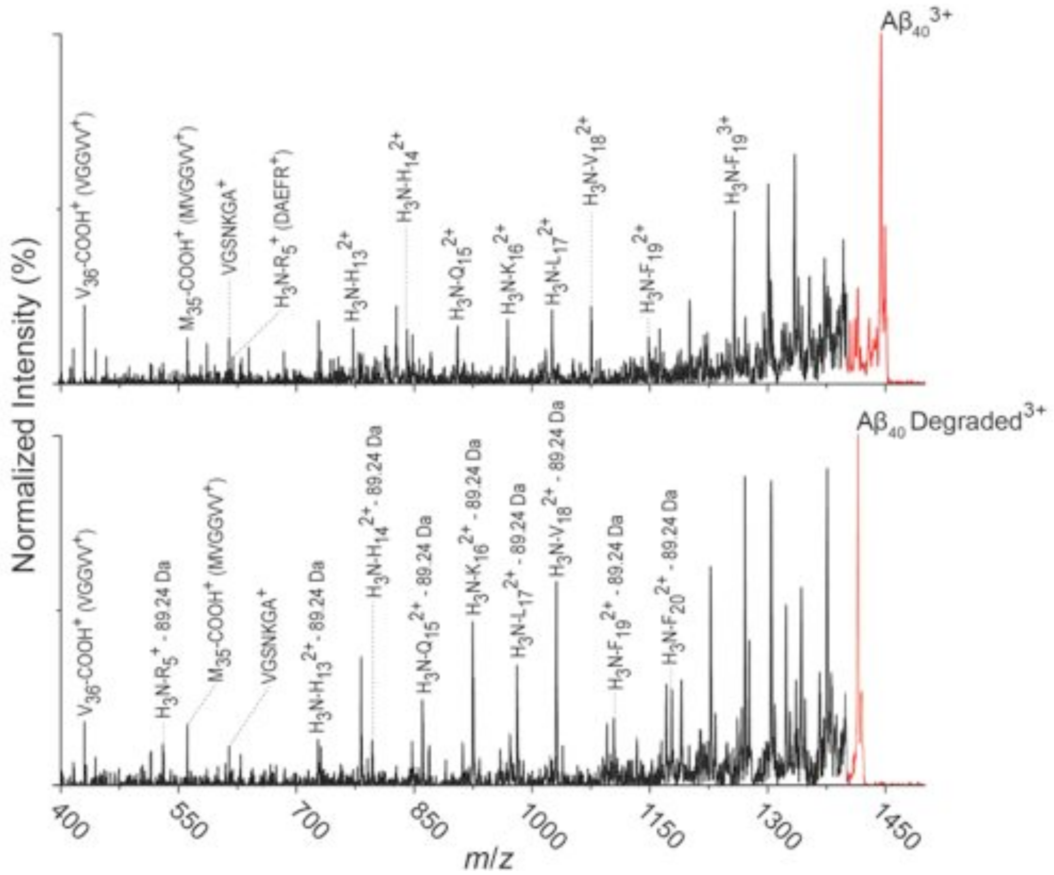

Fig. S3 Comparison of tandem MS/MS sequencing of Aβ$_{40}$ (top) and the identified Cu(II)–**L2-b**-dependent chemical modification product (bottom), using the quadrupole isolated 3+ charge state (trap collision energy 90 V). These data support that in the presence of both Cu(II) and **L2-b**, the Aβ amino acid sequence is chemically modified at a position between the primary amine on the N-terminus and R5, resulting in a calculated mass shift of 89.24 Da. Aβ$_{40}$ F4A MS data, shown below (Fig. S4), support the conclusion that F4 is not the target of this modification. Spectra depicted in black represent a 5X base signal magnification.

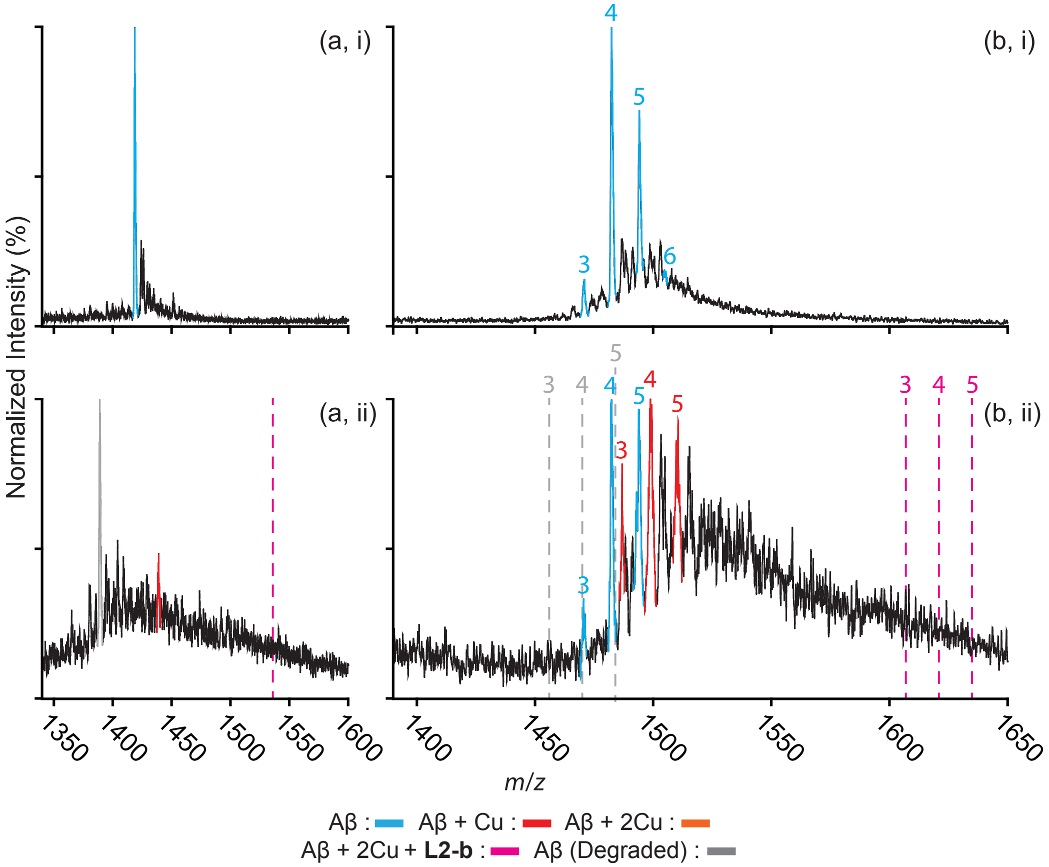

Fig. S4 MS analyses of Aβ_{40} F4A and acetylated Aβ_{40} in the presence and absence of both **L2-b** and Cu(II). (a) Data for the 4+ charge states of Aβ_{40} F4A (18 μM) incubated with (ii) and without (i), **L2-b** (80 μM) and Cu(II) (40 μM). Data support the conclusion that F4 is not required to promote the **L2-b** and Cu(II) dependent chemical modification observed (gray signal, ii). (b) Data for the 4+ charge states of acetylated Aβ_{40} (18 μM) incubated with (ii) and without (i), **L2-b** (80 μM) and Cu(II) (40 μM). While Cu(II) binding is still observed (red signals, ii), data support the conclusion that at least one Aβ_{40} primary amine is required to stabilize the interaction between Aβ_{40} and **L2-b,** as neither the bound nor chemically modified species is observed (pink dashed lines indicate location of the expected m/z values for bound states). Numerals shown above the MS peaks indicate the number of acetyl modifications detected for a given peak. Mass analysis supports a range of 3 to 6 acetylated primary amines under our experimental conditions (of a possible 6).[9]

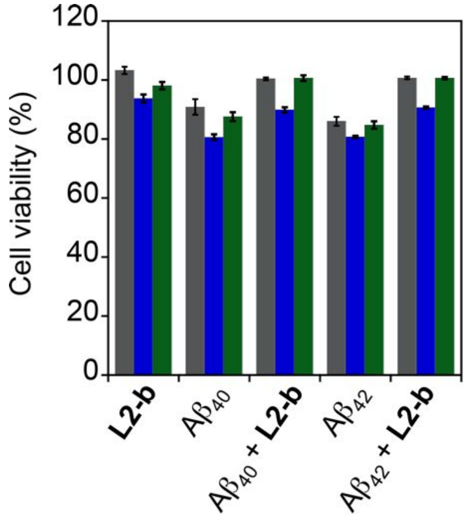

Fig. S5 Influence of **L2-b** on the cytotoxicity of $A\beta_{40}$ and $A\beta_{42}$ in the absence and presence of metal ions. Viability of cells (%) was determined by the MTT assay in the absence (gray) and presence of $CuCl_2$ (blue) or $ZnCl_2$ (green) and calculated relative to that of cells incubated only with 1% v/v DMSO. Error bars represent the standard deviation from three independent experiments. Conditions: [Aβ] = 10 μM; [Cu(II) or Zn(II)] = 10 μM; [**L2-b**] = 10 μM; 24 h incubation.

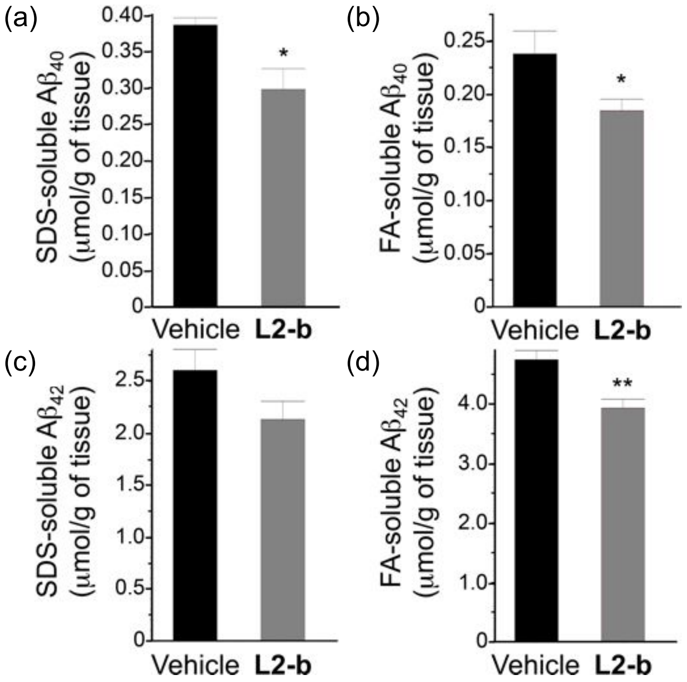

Fig. S6 Effect of consecutive treatments of **L2-b** on the amount of various types of Aβ in the mouse brain. The amounts of SDS/FA-soluble (a and b) $A\beta_{40}$ and (c and d) $A\beta_{42}$ were assessed using ELISA in the whole brain tissues of three-month-old male 5XFAD male mice after three weeks of treatment with vehicle (black bars; n = 5) or **L2-b** (1 mg/kg/day; gray bars; n = 7). Bars denote the levels of Aβ in the different protein fractions, which were calculated from three independent experiments. *$P < 0.05$ or **$P < 0.01$ by one-way ANOVA.
